# Cross-sectional associations of self-perceived stress and hair cortisol with metabolic outcomes and microvascular complications in type 2 diabetes

**DOI:** 10.3389/fpubh.2024.1289689

**Published:** 2024-05-15

**Authors:** Magdalena Buckert, Carmen Streibel, Mechthild Hartmann, Nelly Monzer, Stefan Kopf, Julia Szendrödi, Beate Wild

**Affiliations:** ^1^Department of General Internal Medicine and Psychosomatics, University Hospital Heidelberg, Heidelberg, Germany; ^2^Department of Medicine I and Clinical Chemistry, University Hospital Heidelberg, Heidelberg, Germany; ^3^German Center for Diabetes Research (DZD), Heidelberg, Germany

**Keywords:** psychological stress, hair cortisol, type 2 diabetes, microvascular complications, HbA1c

## Abstract

**Introduction:**

Increasing evidence supports chronic psychological stress as a risk factor for the development of type 2 diabetes. Much less is known, however, about the role of chronic stress in established diabetes.

**Methods:**

The aim of the current study was to comprehensively assess chronic stress in a sample of 73 patients with type 2 diabetes and 48 non-diabetic control participants, and to investigate associations with indicators of glycemic control (HbA1c), insulin resistance (HOMA-IR), β-cell functioning (C-peptide), illness duration, and the presence of microvascular complications. Chronic stress was measured using questionnaires [the Perceived Stress Scale (PSS), the Screening Scale of the Trier Inventory of Chronic Stress (SSCS), the Perceived Health Questionnaire (PHQ) as well as the Questionnaire on Stress in Patients with Diabetes—Revised (QSD-R)]; hair cortisol was used as a biological indicator.

**Results:**

We found that patients with type 2 diabetes had higher levels of hair cortisol in comparison to the control group (*F*(1,112) = 5.3; *p* = 0.023). Within the diabetic group, higher hair cortisol was associated with a longer duration of the illness (*r* = 0.25, *p* = 0.04). General perceived stress did not show significant associations with metabolic outcomes in type 2 diabetes patients. In contrast, higher diabetes-related distress, as measured with the QSD-R, was associated with lower glycemic control (*r* = 0.28, *p* = 0.02), higher insulin resistance (*r* = 0.26, *p* = 0.03) and a longer duration of the illness (*r* = 0.30, *p* = 0.01).

**Discussion:**

Our results corroborate the importance of chronic psychological stress in type 2 diabetes. It appears, however, that once type 2 diabetes has developed, diabetes-specific distress gains in importance over general subjective stress. On a biological level, increased cortisol production could be linked to the course of the illness.

## 1 Introduction

Psychological stress is increasingly recognized as a risk factor for the development of type 2 diabetes. On the other hand, living with a diagnosis of diabetes is also known to be stressful as it requires compliance with the treatment regimen and is related to both fear of hypoglycemia and worries about the future. These aspects are captured by the concept of diabetes distress (1). Higher levels of diabetes distress have been linked to higher HbA1c cross-sectionally [e.g., (2, 3)], whereas results of prospective studies are mixed (4, 5). Conversely, psychological interventions that target diabetes distress were able to improve the HbA1c (6, 7). Higher levels of diabetes distress have also been found in diabetes patients with complications in some (8, 9), but not all studies (10).

Endocrine dysregulation (specifically regarding cortisol secretion) has been proposed as a biological mechanism linking chronic psychological stress and diabetes (11). For instance, elevated levels of cortisol stimulate gluconeogenesis, lipolysis with the release of free fatty acids as well as the accumulation of visceral fat (11). Hair cortisol concentration (HCC) is used to assess long-term integrated cortisol production, and thus is unbiased by circadian variations of cortisol secretion. In fact, HCC has been found to be higher among patients with diabetes compared to non-diabetic participants (12, 13).

Less is known, however, about the relationship between chronic psychological stress, HCC, and diabetic complications in established type 2 diabetes. However, illness burden in diabetes stems mainly from complications; complications increase not only morbidity, but also mortality (14). It should be noted that strict glycemic control cannot prevent the development of diabetic complications (15). It is therefore of the utmost importance that the determinants of diabetic complications be uncovered.

Diabetic complications comprise micro- and macrovascular damages. Macrovascular complications include coronary artery disease, peripheral arterial disease, and stroke (16). Microvascular damages are diabetes-specific and include diabetic nephropathy, neuropathy, and retinopathy. Endothelial dysfunction has been uncovered as a main determinant of diabetic vascular complications (17). In pre-diabetic patients, increased inflammation and oxidative stress have been reported to impair endothelial function (18). Interestingly, chronic stress also promotes a pro-inflammatory state as well as oxidative stress, and may thus affect endothelial function through the same mechanisms (19). Another mechanism affecting blood vessels involves insulin resistance, increasing cardiovascular risk even without a diagnosis of diabetes (20). Insulin resistance might also be increased by chronic psychological stress through several molecular pathways including the hypothalamus-pituitary–adrenal (HPA) axis and the autonomic nervous system, as well as other physiological systems (11, 21).

The aim of the current study was to investigate the association of both chronic psychological stress and diabetes distress with (a) diabetes-specific metabolic outcomes, and (b) the presence of diabetic microvascular complications. Psychological stress was assessed comprehensively by using questionnaire data and hair cortisol as a biological indicator of chronic stress. HbA1c, the HOMA index, and levels of C-peptide were measured as metabolic outcomes.

## 2 Method

### 2.1 Sample

The study sample consisted of 127 participants who were recruited within the context of a larger study through the diabetes outpatient clinic of the University Hospital of Heidelberg, announcements in libraries, pharmacies and other public places, as well as newspaper advertisements. Eligibility for participation in the study was based on the following: that participants were between 40 and 80 years of age, had sufficient speech comprehension, and reading/writing ability without assistance. Smoking more than 10 cigarettes per day, use of illegal drugs, and regular consumption of more than three alcoholic beverages per day were also exclusion criteria [for further details see (22, 23)]. Control participants were eligible for inclusion if they reported no current or past diagnosis of diabetes and no other current chronic or acute medical condition. The control participants were matched to the patients on the basis of mean age and gender distribution, ensuring that the two groups were similar in this regard. Six persons were excluded from analysis due to non-compliance to the study requirements (*n* = 4) and having had bariatric surgery (*n* = 2). Of the remaining 121 participants, 73 had a physician-confirmed diagnosis of type 2 diabetes. Of these, 57 had microvascular complications—defined as described (Table 1 provides the characteristics of the sample).

**Table 1:** Characteristics of the sample.

	T2D with complications	T2D without complications	T2D total	Controls	*p* ^a^	*p* ^b^
N	57	16	73	48		
Gender (% female)	36.8%	31.3%	35.6%	41.7%	0.502	0.680
Age (years)	66.4 (7.3)	63.4 (9.7)	65.7 (7.9)	63.1 (7.8)	0.073	0.184
Illness duration (years)	15.3 (10.9)	7.7 (7.6)				0.014
BMI (kg/m^2^)	30.4 (5.3)	28.3 (6.9)	29.9 (5.7)	26.6 (6.2)	<0.003	0.212
HbA1c (%)	7.3 (0.9)	7.0 (1.3)	7.2 (1.0)	5.4 (0.4)	<0.001	0.308
HOMA-IR	5.8 (4.3)	3.5 (3.4)	5.3 (4.2)	1.9 (1.6)	<0.001	0.004
C-Peptide (ng/ml)	3.2 (2.0)	2.6 (1.5)	3.1 (1.9)	2.2 (1.2)	0.026	0.485

Data are given as mean (standard deviation). T2D = Patients with type 2 diabetes.

a Comparison of patients with type 2 diabetes (total) with controls.

b Comparison of patients with type 2 diabetes with complications and patients with type 2 diabetes without complications.

### 2.2 Procedure

The study was approved by the local ethics committee of the University Hospital of Heidelberg (S-019/2017). All examinations were conducted at the University Hospital of Heidelberg, starting between eight and ten a.m. Participants were examined after an overnight fast. After providing written informed consent, an indwelling catheter was inserted; they then completed several questionnaires including those assessing chronic subjective stress (please see below). Approximately one hour after the beginning of the study, blood samples were taken. Later on a hair sample was collected [additional details regarding the study protocol are described elsewhere (22)].

### 2.3 Definition of complications

In patients with type 2 diabetes, information regarding retinopathy, nephropathy, and peripheral neuropathy was obtained from their medical record, or if not available, by an additional medical examination performed in the diabetes outpatient clinic of the University Hospital of Heidelberg. The examination included funduscopy, assessment of the albumin-creatinine ratio in urine, and questionnaires about neuropathy [see (22), for details].

### 2.4 Metabolic parameters and serum cortisol

Glycated hemoglobin (HbA1c), homeostatic model assessment of insulin resistance (HOMA-IR), C-peptide, and serum cortisol were analyzed in the accredited central laboratory of the University Hospital of Heidelberg using standard operating procedures according to the manufacturers’ instructions. Whole blood samples were centrifuged at 3,500 g for ten minutes. Plasma and serum samples were either analyzed directly or stored at −20°C before analysis. C-peptide was analyzed on a Siemens Immulite 2000 Immunoassay System (reagents kit L2KPEP2). Cortisol and insulin were analyzed on a Siemens ADVIA Centaur XPT Immunoassay System (reagents kits 04344187 and 02230141, respectively). HOMA-IR was calculated according to the formula insulin (mU/l) × glucose (mg/dl)/405. HbA1c was analyzed by HPLC (Variant II Turbo, Bio-Rad).

### 2.5 Hair cortisol

A small, three centimeter long sample of scalp-near occipital hair was collected and sent to the laboratory of Prof. Kirschbaum (Dresden, Germany) for analysis of hair cortisol concentration (HCC). Given an average hair growth rate of 1 cm/month, this reflects an integrated retrospective measure of the cortisol output of the past three months (24). HCC was determined with a commercially available immunoassay with chemiluminescence detection (CLIA, IBL-Hamburg, Germany), employing the protocol of Davenport et al. (25) (the intraassay and interassay-coefficient of variance of this assay is below 8%). In conjunction with hair sampling, a protocol was filled in to assess hair washing frequency and hair treatment (i.e., hair dying, hair coloring, or permanent wave). For statistical analysis, a dichotomous variable for hair treatment (yes/no) was built.

### 2.6 Questionnaires

For the assessment of psychosocial stress, various questionnaires were applied that measure different aspects of stress. All questionnaires show a good reliability and validity (26–28). The German 14-item perceived stress scale PSS (29) was used to assess the frequency of situations such as feeling nervous and stressed, or a perception of having control over one’s life within the past month. The screening scale for chronic stress SSCS/TICS-12 (28) covers a larger time frame; it includes 12 items that assess how often situations of worry, overload, and lack of social recognition have occurred within the last three months. Furthermore, the 10-item stress module of the German version of the Prime MD Patient Health Questionnaire (PHQ) (30) was used. The extent of impairment due to problems in the areas of personal health, social as well as working life, financial status, and past burdensome experiences within the last four weeks was assessed. This stress module has proven to be sensitive to change in patients with diabetes (26).

In addition, the Questionnaire on Stress in Patients with Diabetes—Revised QSD-R (31) was sent to patients with diabetes mailed at least one week before the examination, completed at home. The daily burden of diabetes disease and therapy was assessed by 45 items constituting eight scales: leisure time, work, partner, treatment regimen, hypoglycemia, physical complaints, doctor-patient relationship, and depression/fear of the future. This questionnaire was applied to evaluate the differences between diabetes patients with and without complications in their self-perceived illness related stress.

### 2.7 Statistical analysis

Metabolic parameters as well as hair cortisol were positively skewed and therefore log transformed for group comparisons. Groups were compared using analysis of (co-)variance (AN(C)OVA) in two sets of analyses: First, patients with diabetes type 2 were compared to control participants. Second, within the patient group, those with microvascular complications were compared to those without such complications. To compare type 2 diabetes patients and control participants, gender was included as a second factor to investigate potential moderating effects. Potential covariates were tested first and included only in further analyses in the event that a significant effect was apparent. In regard to stress questionnaires and HCC, age was tested as covariate. A significant association was obtained only for the SSCS score. Regarding HCC, hair washing frequency and hair treatment were additionally considered as potential covariates, but yielded no significant influences. In addition, QSD-R score and subscales were investigated using non-parametric tests (Mann–Whitney-U) to account for deviations from normality. Further, associations between stress questionnaires, HCC, and metabolic parameters were tested by Spearman’s Rho. Due to sporadic missing data, sample size for analyses ranged between 115 and 121 for the entire sample, and between 66 and 73 for the patient sample; serum cortisol data were available for 112 participants. All analyses were carried out using IBM SPSS Statistics 26.

## 3 Results

### 3.1 Hair cortisol

**Figure 1:**
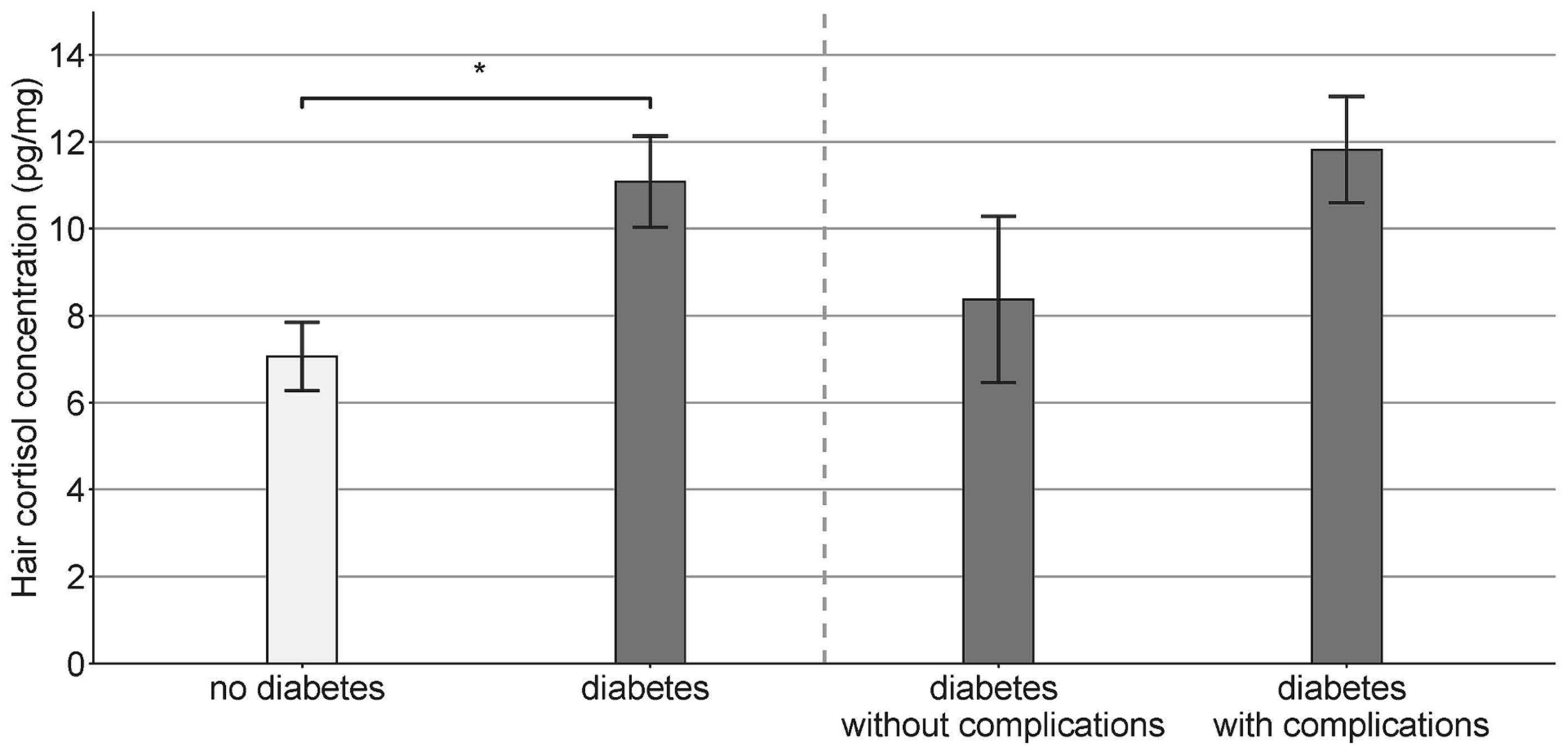
Hair cortisol concentration (mean ± SEM) in patients with type 2 diabetes with and without complications and in the non-diabetic control group (* *p* < 0.05).

HCC was significantly higher among patients with type 2 diabetes compared to control participants (*F*(1,112) = 5.3; *p* = 0.023; Figure 1). Within the patient group, the difference between those with complications compared to those without complications did not, however, reach statistical significance (*F*(1,68) = 3.3, *p* = 0.075; Figure 1).

### 3.2 Stress questionnaires

A significant interaction effect of group and gender emerged for the SSCS (*F*(1,115) = 9.7; *p* = 0.002). Women with diabetes had higher SSCS values compared to female control participants (*F*(1,43) = 3.9; *p* = 0.056), while in men the opposite pattern was observed (*F*(1,71) = 4.5; *p* = 0.037). For the PSS scores the interaction term of group and gender did not reach significance (*F*(1,113) = 2.9; *p* = 0.093).

Regarding PHQ stress scores, patients with type 2 diabetes had slightly (but not significantly) higher values than control participants, independent of gender (*F*(1,116) = 3.3; *p* = 0.071). Regarding PSS, SSCS, PHQ, or QSD-R values within the type 2 diabetes patient group, there were no differences between patients with complications and those without complications. However, regarding work (*p* = 0.047), and physical complaints (*p* = 0.029), an exploratory analysis for each of the QSD-R subscales revealed that type 2 diabetes patients with complications had significantly higher scores than those without.

### 3.3 Correlations

In the entire study sample HCC was not significantly related to baseline cortisol. Furthermore, HCC was not significantly associated with any of the subjective measures of chronic stress, neither in type 2 diabetes patients nor in the control group. Within type 2 diabetes patients, higher HCC was associated with longer illness duration (*r* = 0.25, *p* = 0.04) while associations with metabolic outcomes were weak (Table 2).

**Table 2:** Correlations between indicators of chronic stress and metabolic outcomes in patients with type 2 diabetes.

	HCC	PSS	SSCS	PHQ	QSD-R	HbA1c	HOMA-IR	C-Peptide
PSS	−0.07							
SSCS	0.11	0.56^**^						
PHQ	−0.09	0.32^**^	0.56^**^					
QSD-R	0.01	0.20	0.57^**^	0.60^**^				
HbA1c	0.12	0.02	0.20	0.13	0.28^*^			
HOMA-IR	0.21	−0.05	0.13	0.09	0.26^*^	0.51^**^		
C-Peptide	0.13	0.04	0.04	−0.01	0.06	0.05	0.47^**^	
Illness duration	0.25^*^	−0.07	0.11	0.17	0.30^*^	0.27^*^	0.23	−0.18

* *p* < 0.05, ** *p* < 0.01; *N* between 66 and 72. HCC = hair cortisol concentration; PSS = perceived stress scale; SSCS = screening scale for chronic stress; PHQ = patient health questionnaire; QSD-R = questionnaire on stress in patients with diabetes – revised; HbA1c = glycated hemoglobin; HOMA-IR = homeostatic model assessment of insulin resistance.

Additionally, QSD-R scores were positively associated with the HbA1c (*r* = 0.28, *p* = 0.02) and the HOMA index (*r* = 0.26, *p* = 0.03) as well as illness duration (*r* = 0.30, *p* = 0.01). No other significant correlations were seen in type 2 diabetes patients between subjective diabetes-unspecific stress scales and metabolic outcomes, including in illness duration (Table 2).

## 4 Discussion

Hair cortisol levels in patients with type 2 diabetes were significantly higher compared to control participants and positively associated with illness duration. Regarding questionnaire data, only diabetes distress was associated with markers of illness severity. Thus, the link between chronic stress and the course of diabetes appears to be complex, depending on diabetes outcome and the specific stress measure.

Cortisol concentrations in hair followed the expected pattern, i.e., they were highest in type 2 diabetes patients with complications, followed by type 2 diabetes patients without complications, and lowest among control participants. Higher hair cortisol concentrations in patients with type 2 diabetes compared to controls have been reported previously (12). However, previous studies have rarely used hair cortisol to investigate the relationship with markers of illness severity. Lehrer et al. (32) report a positive association of hair cortisol and HbA1c among African-American type 2 diabetes patients. Similar results were obtained by Stalder et al. (33) in a sample of Caucasian participants. However, the latter study did not focus specifically on patients with diabetes. While we did not obtain a significant association between HCC and HbA1c, HCC was related to illness duration.

To date, hair cortisol levels have not yet been investigated in relation to the presence of diabetic microvascular complications. Zhang et al. (34) found higher serum cortisol levels in T2D patients with microalbuminuria; higher serum and urinary free cortisol levels were also reported by Chiodini et al. (35) for T2D patients with chronic diabetic complications including nephropathy, neuropathy, retinopathy as well as silent macroangiopathy. In summary, we could speculate that cortisol might be causally involved in the progress of the illness including the development of diabetic complications. However, due to the correlational nature of our study, influences of other factors on hair cortisol levels cannot be excluded [cf. (36), for a meta-analysis of potential factors]. For example, the immune system and the HPA axis are strongly connected; a large study has recently shown that hair cortisol concentration was associated with markers of obesity as well as indicators of low-grade inflammation (37). Besides measurement issues associated with subjective statements such as low sensitivity, such influences might also contribute to the well-known lack of correlation between self-perceived stress and hair cortisol levels (36); this was also observed here.

In addition, it should be noted that cortisol—despite undoubtedly being an important factor— is not the sole mediator of the biological stress response in the context of diabetes (11, 21), and hair cortisol is only one indicator of HPA axis activity (38), specifically reflecting cortisol output over a longer period of time (24). Furthermore, cortisol action in the periphery is locally modulated by enzymes in the target tissue. For example, 11β-hydroxysteroid-dehydrogenase 1, which converts inactive cortisone into active cortisol, is increased in the adipose tissue of obese humans (38). Thus, the relationship between stress and diabetes is multifaceted, and each study can only investigate pieces of the complex interplay. For instance, in addition to hormonal assessments, it might be interesting in further studies to also use multimodal sensing and its integration via the Internet of Things and machine learning to continuously monitor stress in real-time (39, 40), possibly along with metabolic parameters (41).

Higher levels of subjective stress were reported by patients with type 2 diabetes compared to control participants depending on gender. This is in line with previous cohort studies which also, quite frequently, found moderating effects of gender on the association of subjective stress and diabetes incidence. Surprisingly, we found no significant differences between diabetes type 2 patients—those with and those without microvascular complications—with respect to questionnaires assessing general (i.e., diabetes-unspecific) perceived stress. In addition, diabetes-unspecific subjective stress was not related to diabetes-associated metabolic outcomes in the context of the patient sample. In contrast, diabetes-specific distress was associated with glycemic control and illness duration.

The lack of association between general perceived stress and metabolic outcomes is in line with a previous study reporting that only diabetes distress, but not the PHQ or serious psychological distress was related to the HbA1c in a sample of patients with type 2 diabetes (42). We can therefore infer that in patients with type 2 diabetes, glycemic control is not associated with the self-assessment of everyday life stress. One possible explanation is that patients with this condition may be biased in their perception of stress, making them more sensitive to disease-specific stressors. Another inference is that once diabetes has developed, general psychological stress takes a back seat whereas diabetes-specific aspects come to the fore. It should be noted, however, that longitudinal studies will be needed to test this hypothesis. In addition, causality cannot be inferred from the current results. It is conceivable that diabetes and its associated complications result in diabetes distress; on the other hand the effectiveness of psychological interventions indicates the opposite. Thus, the link between diabetes distress and diabetes (complications) is, most likely, bi-directional.

The major strength of the current study is that stress was assessed comprehensively, using various questionnaires as well as hair cortisol. In addition, type 2 diabetes patients differed with respect to diabetes duration as well as the presence of microvascular diabetic complications. This allowed us to investigate associations of stress with illness severity as reflected by several diabetes-associated metabolic outcomes. However, some limitations should be noted. First, there were only a few participants without microvascular complications, thus limiting the power of the comparison tests and analyses of potentially moderating factors (43). It could be due to the insufficient power that the difference in hair cortisol between patients with and without complications narrowly failed to reach significance. However, it was particularly difficult to find diabetes patients without complications; consequently, future studies may be enhanced by allocating additional resources to the recruitment of this specific subgroup. Second, because a different measurement method was used we could not categorize individuals in groups with either high or low stress levels according to given cut-off values (44). Taking into account that associations between self-perceived stress and HCC seem to be stronger within highly stressed persons (45), it could be interesting to include a specifically selected, high-stress subsample in future studies. Third, our study may have been subject to various biases. A selection bias may have been occurred due to the recruitment of diabetes patients in a University Hospital setting. Also, the participants’ self-assessment of stress could be subject to a memory bias. However, we would rule out the reverse causality bias because all our inferences refer to possible associations and not to causal pathways.

In conclusion, our results support the importance of psychological stress in type 2 diabetes. However, diabetes-unspecific subjective stress appears not to play a major role in relation to the severity of the illness as reflected by glycemic control, insulin resistance, β-cell function, and the presence of microvascular complications. Nevertheless, on a biological level, cortisol production could be linked with diabetes outcomes. Reducing cortisol levels might therefore be important also after the diagnosis of diabetes has been received. In addition, psychological interventions could benefit from explicitly addressing diabetes-specific topics. To establish the chronological order of these associations, longitudinal studies are warranted.

## Data availability statement

The raw data supporting the conclusions of this article will be made available by the authors, without undue reservation.

## Ethics statement

The studies involving humans were approved by the ethics committee of the University of Heidelberg [S-019(2017)]. The studies were conducted in accordance with the local legislation and institutional requirements. The participants provided their written informed consent to participate in this study.

## Author contributions

MB: Conceptualization, Formal analysis, Funding acquisition, Project administration, Writing – original draft, Writing – review & editing. CS: Formal analysis, Investigation, Visualization, Writing – review & editing. MH: Conceptualization, Funding acquisition, Supervision, Writing – review & editing, Project administration. NM: Investigation, Writing – review & editing. SK: Methodology, Resources, Writing – review & editing. JS: Resources, Writing – review & editing. BW: Conceptualization, Funding acquisition, Project administration, Supervision, Writing – review & editing.
